# Pancreatic enzyme replacement therapy can improve infection level nutrition condition and prognosis of patients with sepsis

**DOI:** 10.29219/fnr.v69.12746

**Published:** 2025-07-21

**Authors:** Li Zhao, Sheng Wang

**Affiliations:** 1 Department of Critical Care Medicine, Taikang Tongji (Wuhan) Hospital, Wuhan, 430050, Hubei Province, P.R. China; 2 Department of Critical Care Medicine, Shanghai Tenth People’s Hospital, Tongji University, Shanghai, 200072, P.R. China

**Keywords:** exocrine pancreatic insufficiency, pancreatic enzyme replacement therapy, malnutrition, sepsis

## Abstract

**Objective:**

This study aimed to investigate the effects of pancreatic enzyme replacement therapy (PERT) on infection level, nutrition condition and prognosis of patients with sepsis.

**Method:**

According to the fecal elastase-1 (FE1) level, 68 sepsis patients who were diagnosed with pancreatic exocrine insufficiency (PEI) from 2014.11 to 2015. 12 in our hospital were randomly divided into two groups: regular nutritional support (RNS) group or PERT group. A total of 15 patients were dropout for various reasons.

Finally, 25 patients were enrolled in PERT group and 28 in RNS group. APACHEII score, SOFA score, inflammatory biomarkers including C-reaction protein (CRP), white blood cell (WBC), procalcitonin (PCT), nutrition markers including prealbumin (PA), transferrin (TFN), retinol binding protein (RBP), creatinine/height index (CHI) were recorded at the day 1 (D1), day7 (D7) and day14 (D14) since they were admitted in ICU. These data were compared between and within the two groups chronologically. Also, the duration of vasoactive drug using (DVAD), mechanical ventilation (DMV), length of stay in ICU (LOS) and survival rate within 14 days were compared between the two groups.

**Results:**

There were no differences in general information (Age and gender) between PERT and RNS groups. Compared with the RNS group, CRP, WBC and PCT declined significantly at D14 in the PERT group. Especially, CRP declined significantly over time in both groups. In addition, compared with the RNS group, in the PERT group at D14, nutrition markers, including PA, TFN, RBP and CHI increased significantly, APACHEII score and SOFA score decreased significantly. And DVAD, DMV and LOS were significantly shortened in PERT group, but the survival rate within 14 days was not significantly changed.

**Conclusion:**

The PERT can improve infection level, nutrition condition and prognosis of patients with sepsis. And the underlying mechanism may be related to improve pancreatic exocrine insufficiency of these patients.

## Popular scientific summary

- PERT can improve infection level, nutrition condition and prognosis of PEI patients with sepsis to a certain extent. And the underlying mechanism may be related to improve pancreatic exocrine insufficiency of these patients.

Pancreatic exocrine insufficiency (PEI) is defined as an insufficient amount of pancreatic enzymes to maintain a normal digestion, which is associated with nutritional deficiencies leading to osteoporosis, low-trauma fractures, sarcopenia and increased mortality (1, 2). PEI is common in patients with pancreatic disease, but the latest research suggests that patients, especially in critical unit (ICU) with severe diseases, may suffer from pancreatic injury in the absence of other plausible causes of pancreatic dysfunction and is also common in critically ill people without pre-existing pancreatic disease (3, 4).

In China, the overall hospital mortality rate of sepsis was 20.6%, but the mortality rate was close to 50% in a Subdistrict of Beijing (5). Although the clinical and basic research of sepsis has made great progress and the mortality of severe sepsis has declined, but severe sepsis remains a big burden of society (6). Furthermore, epidemiological data suggest that more than 50% of severe ill adult patients without pancreatic diseases have complicated PEI, and nearly one-fifth of them have severe PEI (7).

PEI diagnosis is predominantly based on clinical findings and the presence of underlying disease. As these symptoms are non-specific, they are often failed to attract enough attention of clinicians, then failed to be detected and treated timely. As a result, PEI is frequent but underdiagnosed and undertreated (8), which could lead to malnutrition (9) then to worse prognosis, but could be improved by nutritional support (10–12).

In order to prevent severe ill patients from PEI, pancreatic enzyme replacement therapy (PERT) has been strongly recommended by many national authorities (13). PERT provides immediate symptomatic relief, but the biological basis of this phenomenon remains unclear and warrants further investigation. This study was performed by observing the effects of PERT on patients with sepsis. We find that PERT can improve infection level, nutritional condition, and reduce the DVAD, DMV and LOS of patients with sepsis.

## Materials and methods

### Experiment material

Pancreatin Enteric-coated Capsules (Deimeitong), produced by Abbott laboratories GmbH, is an enteric-soluble pancreatic enzyme ultramicron capsule. Each 0.3 g capsule contains: Pancreatic lipase: 20,000 European Pharmacopoeia Units (Ph. Eur.); Pancreatic amylase: 16,000 Ph. Eur.; Pancreatic protease: 1,200 Ph. Eur.

### Selection of patients

Patients who were diagnosed with PEI at the ICU of our Hospital from 6/2014 to 12/2015 were prospectively recruited. Fecal elastase-1 (FE1) was tested in all patients promptly after they were enrolled in ICU. PEI was diagnosed as the FE1 was less than 200 μg/g. FE1 were determined by ELISA (Schebo Biotech, Wettenberg Giessen, German).

### Inclusion criteria

1) Over 18 years old without primary pancreatic disease; 2) There are more than two high risk factors of PEI; 3) Severe patients receive enteral nutrition (including basic diet) and are expected to spend more than 7 days in ICU. Primary pancreatic disease refers to acute pancreatitis, chronic pancreatitis, postpancreatectomy, cystic fibrosis of the pancreas and pancreatic ampulla abdominal tumor. Risk factors refer to gastrectomy, enterectomy, Zollinger-Ellison syndrome, inflammatory bowel disease, Crohn’s disease, celiac disease, diabetes (fasting glucose > 7 mmol/L), anemia (hemoglobin < 80 g/L), hypotension (mean arterial pressure < 70 mmHg) lasting over or equal to 1 h, shock (systolic blood pressure < 90 mmHg), sepsis, craniocerebral trauma, cerebral hemorrhage, cerebral infarction, respiratory failure (PaO2 < 60 mmHg), mechanical ventilation, continuous renal replacement therapy, hyperlacticemia (blood lactic acid > 2 mmol/L), hyperbilirubinemia (total bilirubin > mol/L), hyperlipidemia (triglycerides > 1.7 mmol/L), obesity (BMI > 30 kg/m2).

### Exclusion criteria

1) Younger than 18 years old; 2) LOS less than 7 days or die within 7 days in ICU; 3) pregnancy or breastfeeding; 4) primary pancreatic disease; 5) the time of enteral nutritional intolerance more than 3 days during the study period; 6) fecal specimens cannot be collected within 72 h after enteral nutrition begins; 7) patients or authorized immediate family members declined to participate in the study.

## Methods

### Ethics statement

The study was carried out in compliance with the guidance suggestion of Human Ethics Committee of Tongji University (Permit number: SYXK [Guangdong] 2010-0104). The protocol was approved by the ethics committee of Tongji university (Permit number: 2011-54). All therapeutic measures were performed in accordance with the sepsis guidelines, and all efforts were made to minimize suffering. All patients received detailed information and provided written informed consent by themselves or their immediate family members if patients were not able to make informed consent.

### Sample and clinical data collection

This prospective double-blind randomized parallel study was conducted in Department of Critical Care Medicine, our hospital. All the patients were divided into RNS group or PERT group by using random number method. All the patients received the regular treatment such as anti-infection therapy, organ support therapy. But in the PERT group, pancreatic enzyme is added besides the above-mentioned treatments. The diagnosis of sepsis is in accordance with Sepsis 3.0 definition. Venous blood was collected after they were admitted to ICU at D1, D7 and D14 to detect the level of CRP, WBC, PCT, PA, TFN, RBP, creatinine. APACHEII score, SOFA score, LOS, DVAD, DMV and survival rate were also recorded during the study.

### Enteral nutrition prescription

For patients who need tube feeding, resting energy consumption is calculated by using Harris Benedict formula, and is adjusted according to the stress (20% increase) and the degree of activity (10% increase). The amount of energy is prescribed according to the selected prescriptions of enteral nutrition. Stress includes surgery, trauma, burns, or infection. Activity levels include breathing or moving freely.

In PERT group, the dosage of Deimeitong (10,000 U/granule) was prescribed according to the standard that 2,500 U is given to 1 g fat in enteral nutrient solution. For those who can take meals orally, in PERT group, the PERT was carried out as 3 g per meal (mean 10,000 U/capsule, 3 capsules/meal) during the meal.

### Statistical analysis

The χ^2^ test or Fisher’s exact test was used to analyze the general information. The Student’s *t*-test was used to compare infection biomarkers (CRP, WBC, PCT), nutrition markers (PA, TFN, RBP, CHI), APACHEII score, SOFA score, LOS, DAVD and DVM.

Survival rate was estimated by Kaplan-Meier method with the log-rank test. Multivariate analysis was performed using the Cox proportional hazard regression analysis.

Statistical analysis was performed with the SPSS software package (version 22.0, SPSS Institute). *P*-values < 0.05 was considered significant.

## Results

### General information comparison

**Fig. 1:**
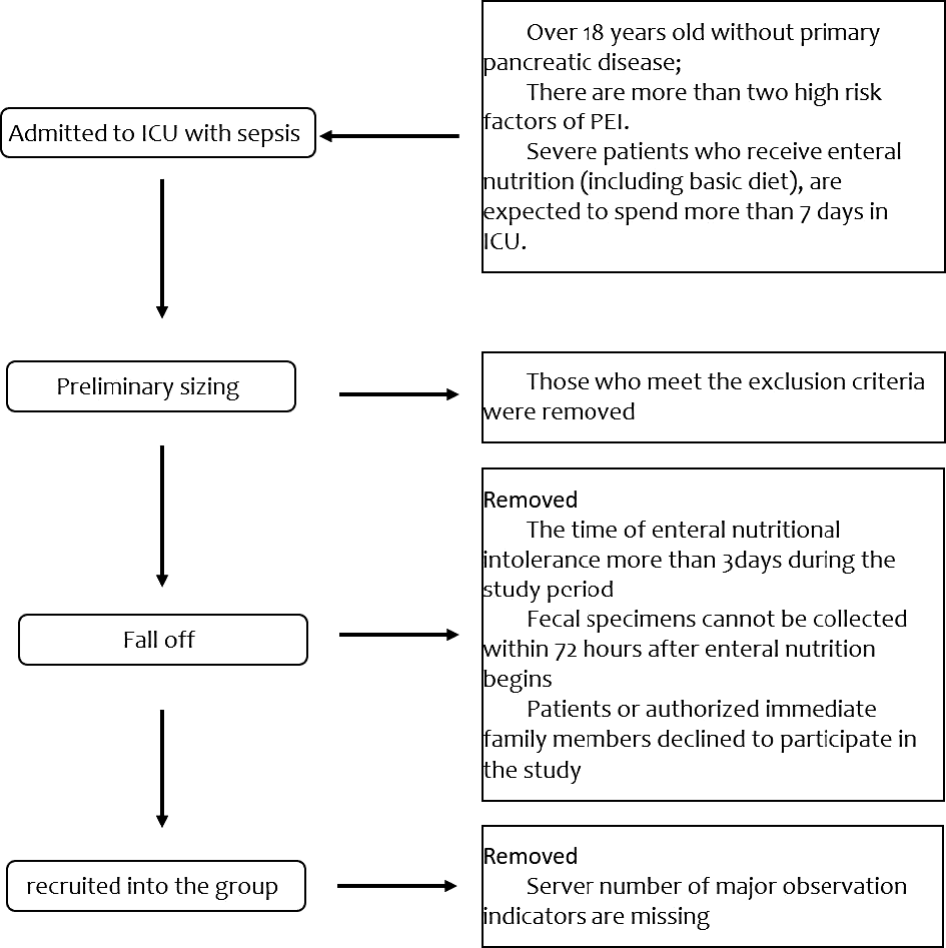
Flowchart of participating patients in the study.

The patient flowchart is shown in Fig. 1. There were 28 patients in RNS group (20 female and eight males), the average age was 71.56 years. While there were 25 patients in PERT group (18 female and seven males) with the average age of 69.33 years. There were no difference in general information (Age, gender, FE1) (*P* > 0.05) (Table 1).

**Table 1:** Comparison of the general information between the RNS and PERT groups

	RNS group	PERT group	*P*
Gender (male/female)	20/8	18/7	0.963
Age (year)	69.33 ± 15.90	71.56 ± 13.55	0.611
FE1 (μg/g)	157.56 ± 24.85	163.56 ± 19.11	0.138

Age was shown as mean ± SD. P-value < 0.05 was considered significant.

### Inflammatory markers at each time point in the two groups

**Fig. 2:**
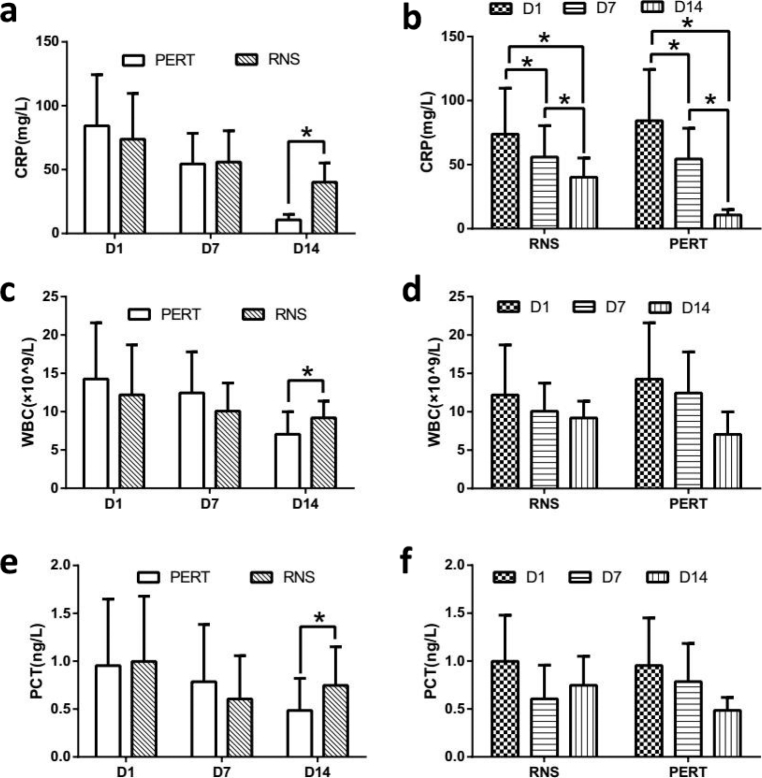
Changes in infection indicators between the two groups. Infection markers (CRP, WBC, PCT) were compared between PERT and RNS groups (Figure 2a–c), or within each group (Figure 2d–f) at different time points. *P < 0.05.

In each group, we saw a declining tendency of CRP, WBC and PCT over time, but only CRP gradually decreased significantly (Fig. 2d) in both groups. Compared with RNS group, CRP, WBC and PCT were all lower significantly at D14 in PERT group (Fig. 2a–d).

### Nutrition markers at each time point in the two groups

**Fig. 3:**
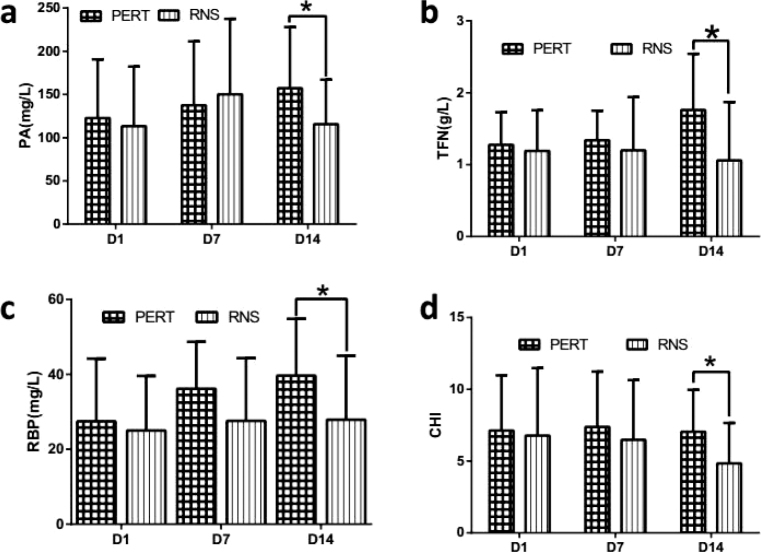
Nutrition markers in two groups. PA (A), TFN (B), RBP (C), and CHI (D) were compared between PERT and RNS groups at different time points. PERT, pancreatic enzyme replacement therapy. RNS, regular nutritional support. PA, prealbumin. TFN, transferrin. RBP, retinol binding protein, CHI, creatinine/height index. The data were presented as the mean ± SD. *P < 0.05.

The difference of the nutrition markers (PA, TFN, RBP and CHI) was not significant in each group at D1 (Fig. 3). After 14 days of PERT, compared with RNS group, all the nutrition markers increased significantly in the PERT group (Fig. 3).

Comparison of APACHEII score and SOFA score at each time point between the two groups.

**Fig. 4:**
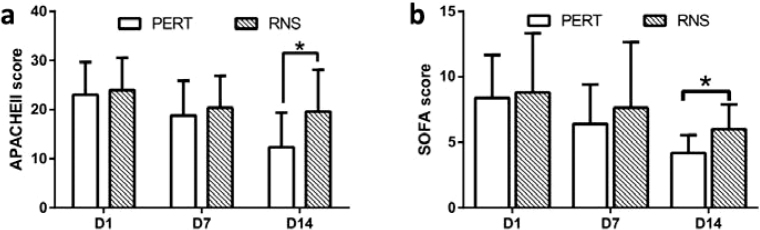
Comparison of APACHEII score and SOFA score at each time point between the two groups. APACHEII score (A) and SOFA score (B) were compared at each time point between PERT and RNS group. PERT, pancreatic enzyme replacement therapy. RNS, regular nutritional support. The data were presented as the mean ± SD. *P < 0.05.

We saw a declining tendency in both groups. Compared with RNS group, APACHEII score and SOFA score were both significantly lower in PERT group at D14 (Fig. 4).

### Prognostic indicators of the two groups

**Fig. 5:**
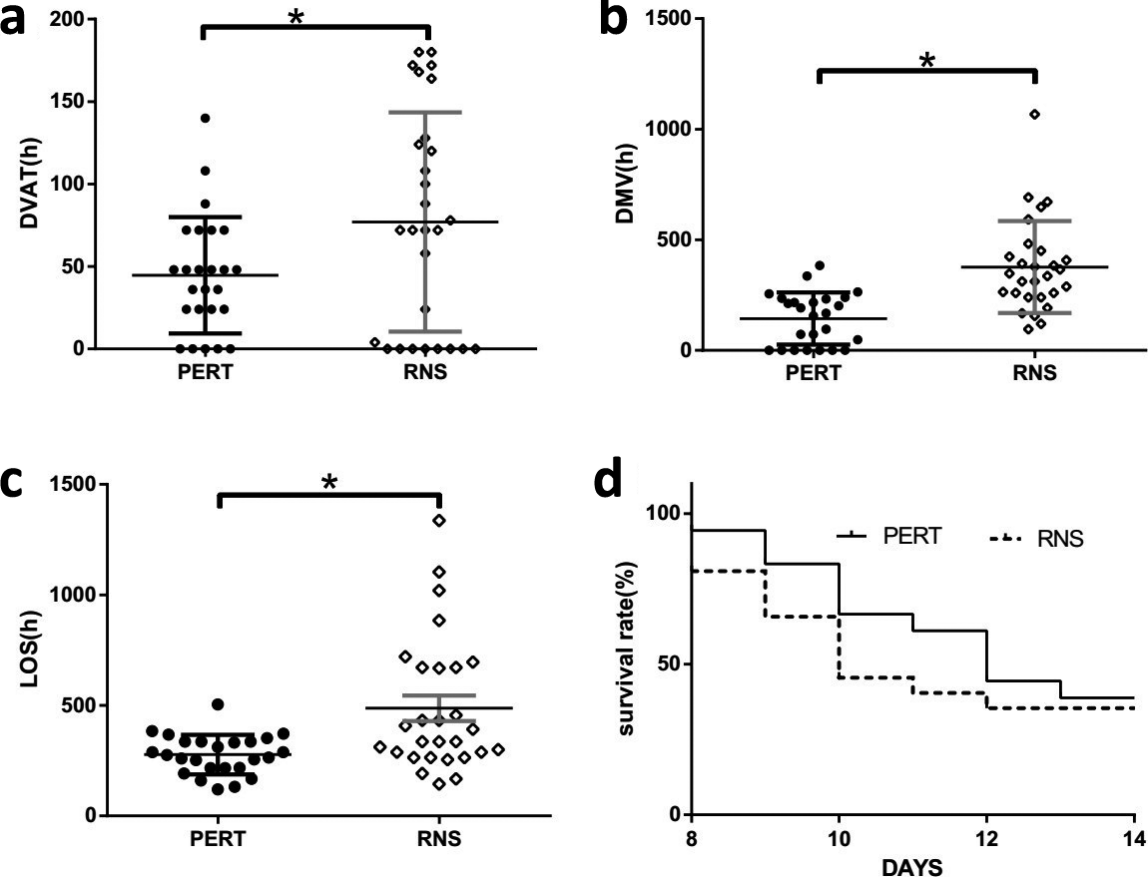
Comparation of prognostic indicators between the two groups. DVAD (A), DMV (B), LOS (C), and survival rate (D) within 14 days were compared between PERT and RNS groups. PERT, pancreatic enzyme replacement therapy. RNS, regular nutritional support. DVAD, duration of vasoactive drug using. DMV, mechanical ventilation. LOS, length of stay in ICU. The data were presented as the mean ± SD. *P < 0.05.

From our study, we can see that the DVAD, DMV, and LOS were significantly shortened, compared with the RNS group (Fig. 5a–c).

Since patients who died within 7 days were excluded from this study, the survival rate of the two patient groups decreased gradually over time, but were not statistically significant different after 14 days (Fig. 5d).

## Discussion

Comparison between sepsis patients and healthy controls shows the secretion of amylase, trypsin and chymotrypsin in sepsis patients decreased significantly (14). We previously screened PEI in critically ill adult patients receiving enteral nutrition, and found several PEI risk factors, like shock, sepsis, diabetes, cardiac arrest, hyperlacticaemia, invasive mechanical ventilation and hemodialysis (7), which are common diseases treated in ICU worldwide. Nowadays, PERT is the main procedure commonly used to improve PEI, however, there is little evidence to evaluate its effects on patients with sepsis.

As we reported for the first time, with FE-1 as the standard, PEI has a high incidence in patients with sepsis (7). Meanwhile, this study also takes FE-1 less than 200 mg/g as cutoff value, and all patients included were high-risk patients with sepsis PEI. By now, there is still no non-invasive gold standard to accurately diagnose PEI in several diseases because of the complication of pathophysiology. FE-1, which has obvious correlation with pancreatic exocrine enzymes, has been proved to be more specific and sensitive than other PEI tests (15). At the same time, FE-1 has the characteristics of low variability and high stability in human feces, and it is resistant against intestinal degradation. Also, FE-1 test needs only a small amount of human stool. So, it is also very suitable for the detection of PEI in sepsis patients. Especially, very low FE-1 level (200 mg/g) significantly correlated with PEI (16, 17).

At present, most studies on PERT focus on observing the improvement of clinical symptoms (bloating, stool consistency, abdominal pain and flatulence) and defecation (stool frequency, consistency and steatorrhea) (18–20). But for mechanical ventilated septic patients, it is difficult to acquire accurate abdominal complaints. Even more difficult, monitoring defecation requires strict diet and long-term collection of feces (21, 22), which is almost impossible for these sepsis patients. As a result, we detected other indicators. Firstly, infection is the second leading cause of death in PEI patients with chronic pancreatitis because of nutritional deficiencies (23). In our study, CRP, WBC and PCT, which are infection indicators, were significantly reduced after 14 days of PERT (Fig. 2), meanwhile, the nutrition indicators, like PA, TFN, RBP and CHI, were also improved (Fig. 3). These were similar with de la Iglesia-Garcia, D’s study in chronic pancreatitis (23). These results suggest that in patients with sepsis-induced PEI, PERT can alleviate inflammation by improving nutritional status. However, it does not necessarily mean that the dose of PERT is enough for the treatment of these patients. Because Min et al. (24) reported that 84.2, 89.1 and 77.8% of PEI patients with chronic pancreatitis still had vitamin A, D and E deficiency after the recommended dose of PERT, respectively, according to the current guidelines, as a result, which could leads to osteopenia, osteoporosis and sarcopenia (24, 25). Osteopenia, osteoporosis and sarcopenia all can result in worse outcomes in various diseases including sepsis (26, 27). Therefore, in the future, researches on sepsis induced PEI needs to further accurately titrate PERT dose and then provide personalized treatment.

PEI induces malnutrition and finally leads to increased risk of hospitalization and reduced survival rate (25). However, in sepsis patients, the study of the impact of PERT on the prognosis related indicators of patients is still very limited. In our study, DVAD, DMV, LOS, APACHEII score and SOFA score of PERT group at D14 statistically reduced in septic PEI patients (Figs. 4 and 5), which indicates PERT can improve prognostic factors in patients with sepsis-induced PEI to some extent. However, there was no significant change in survival rate within 14 days of admission (Fig. 5). In randomized controlled studies of unresectable pancreatic cancer patients, on the one hand, 8 weeks of PERT treatment can help patients maintain a certain weight, alleviate PEI symptoms and improve the quality of life and survival rates (28, 29). On the other hand, PERT did not improve PEI symptoms or significantly improve overall survival (30, 31). For cystic fibrosis children and chronic pancreatitis patients, relatively long-term PERT can significantly improve PEI symptoms and improve life quality (32, 33). We speculate that the short-term and long-term effects of PERT are different in the same disease and the effects of PERT are also different in different diseases. In the long run, whether PERT can improve the survival rate of patients with sepsis PEI, like chronic pancreatitis, cystic fibrosis and other patients, still needs further study to clarify.

## Conclusion

Our study shows that PERT can improve infection level, nutrition condition and prognosis of PEI patients with sepsis to a certain extent. However, further large-scale clinical studies are still needed to confirm this.

## Declarations

### Availability of data and material

The datasets used and/or analyzed during rhis study are available from the corresponding author on reasonable request.
